# Serum Squamous Cell Carcinoma Antigen-Immunoglobulin M complex levels predict survival in patients with cirrhosis

**DOI:** 10.1038/s41598-019-56633-2

**Published:** 2019-12-27

**Authors:** Marco Cagnin, Alessandra Biasiolo, Andrea Martini, Mariagrazia Ruvoletto, Santina Quarta, Silvano Fasolato, Paolo Angeli, Giorgio Fassina, Patrizia Pontisso

**Affiliations:** 1 Department of Medicine, University of Padua, Padua, Italy; 2 Xeptagen S.p.A., Venice, Italy

**Keywords:** Liver cirrhosis, Prognostic markers

## Abstract

Complications of chronic liver diseases – particularly hepatocellular carcinoma (HCC) – are a major cause of mortality worldwide. Several studies have shown that high or increasing levels of serum Squamous Cell Carcinoma Antigen-Immunoglobulin M complex (SCCA-IgM) are associated with development of HCC in patients with advanced liver disease and worse survival in patients with liver cancer. The aim of the present study was to assess, in patients with advanced liver disease, differences in long-term clinical outcomes in relation to baseline levels of serum SCCA-IgM. Ninety one consecutive outpatients with liver cirrhosis of different etiologies, without hepatocellular carcinoma at presentation, were enrolled from April 2007 to October 2012 in a prospective study. For a median time of 127 months, patients were bi-annually re-evaluated. SCCA-IgM complex levels were determined with a validated enzyme-linked immunosorbent assay. The results provided evidence that serum SCCA-IgM is a predictor of overall survival. The best cut-off to discriminate both HCC-free and overall survival rates was 120 AU/mL. Patients with baseline values higher than this threshold showed a substantial increase in both HCC incidence rate and all-cause mortality rate. In conclusion, a single measurement of serum SCCA-IgM helps to identify those patients with liver cirrhosis with increased risks of HCC development and mortality.

## Introduction

Hepatocellular carcinoma (HCC) is a major determinant of cancer-related death worldwide, and the first ranking neoplasm for overall increase in mortality rates in many industrialized and developing countries^1,2^. On a global scale, the high prevalence of HCC is attributable to its common risk factors, which are chronic liver diseases (mainly chronic hepatitis B or C infection, longstanding alcohol abuse and metabolic-associated steatohepatitis) and liver cirrhosis^3^, which itself is the outcome of all progressive liver diseases at end-stage.

A timely diagnosis of HCC – unprecedented or recurrent – is the most important strategy to significantly reduce disease-specific mortality, because treatments at early stages (“zero” and “A” according to the Barcelona Clinic Liver Cancer classification) are more frequently curative, hence associated with better survival and cost-effectiveness in comparison to those performed in intermediate or advanced stages (“B”, “C” and “D”)^4^.

Reports from real-life experience indicate that HCC screening programs are seldom effective^5^. In Western countries, surveillance of the population-at-risk only relies on periodic liver ultrasonography, a suboptimal technique because of low sensitivity^6^.

For hepatocellular carcinoma, several serum biomarkers (i.e. Alpha-fetoprotein and its L3 isoform, Protein Induced by Vitamin K Absence-II, Osteopontin…) are currently available in clinical practice, even if their use is not validated nor regulated. New screening modalities that include novel serum biomarkers, integrative scores, and imaging techniques for early HCC detection are under development or evaluation^7^.

Previous studies indicate that Squamous Cell Carcinoma Antigen (SCCA) overexpression is an early event in hepatic carcinogenesis^8^. SCCA-1 isoform (also known as Serpin B3) is implied in many biological functions, including resistance to apoptosis, induction of cell proliferation and promotion of epithelial-mesenchymal transition^9^. All these complex features may explain why, as described in several cancers of epithelial origin, liver neoplasms that overexpress SCCA also tend to display worse grades of inflammation, anaplasia and invasiveness^10–12^. In this setting, SCCA expression was associated with the production of Transforming Growth Factor Beta (TGF-β)^13^, which is one of the most effective immunomodulators known to promote carcinogenesis^14^.

Many studies on animal models and clinical observations suggest a major role for the immune system in the surveillance against neoplastic-transformed cells^15^. Germline-coded immunoglobulin M (IgM) antibodies are a hallmark component of the so-called cancer immunosurveillance^16^, which is an early phase of the immunoediting process, subsequently characterized by an escape of neoplastic cell from immune clearance^17^.

Through binding to IgM complexes, SCCA isoforms become identifiable in serum^18^. SCCA-IgM is undetectable in healthy controls, while high or increasing levels of serum SCCA-IgM have been associated with the presence of advanced or worsening liver disease and with increased risk of HCC^19,20^. In addition, in patients already diagnosed with HCC, high levels of SCCA-IgM have been described to be a predictor of worse survival^21,22^.

The aim of this prospective study was to evaluate long-term clinical outcomes of patients with liver cirrhosis in relation to their levels of circulating SCCA-IgM at presentation.

## Methods

### Patients

The study population consisted in 91 adult outpatients with known liver cirrhosis of any etiology, consecutively referred to our tertiary care hospital (Regional Referral Center for Liver Diseases of the Padua University Hospital) from April 2007 to October 2012.

The exclusion criteria were: previous or current hepatocellular carcinoma, evidence of hepatic lesions compatible with primary liver neoplasm, potentially active previous or current malignant epithelial neoplasm(s), previous liver transplantation or unavailability of complete clinical information, laboratory tests and imaging data necessary to confirm the diagnosis of liver cirrhosis and its stage at presentation.

All participants expressed their informed consent at the act of enrolment.

None of the patients with Chronic Hepatitis C (CHC) was treated with Direct-Acting Antivirals (DAA) prior to their marketing authorization in Italy (early 2015). Patients with previous or new episodes of cirrhosis decompensation during follow-up were also evaluated for liver transplantation, when appropriated.

In total, 30 patients developed hepatocellular carcinoma during follow-up. These patients were treated accordingly to the Barcelona Clinic Liver Cancer management flowchart and considered for liver transplantation, when fulfilling the Milan criteria^4^. For 20 of these patients (66.7%), the first-line treatment consisted in a loco-regional therapy, being in 10 cases Transcatheter Arterial Chemo-Embolization (TACE), in 9 cases Radio-Frequency Ablation (RFA), in one case Percutaneous Ethanol Injection (PEI) and in one case surgical resection. On the other hand, 10 of these patients (33.3%) were not suitable for a loco-regional therapy at the time of diagnosis, hence were directly assigned to the liver transplantation pathway or to systemic chemotherapy, when indicated.

Demographic, clinical and biochemical profile of the study sample is listed in Table 1.

**Table 1:** Baseline characteristics and main outcomes of the study population.

Variable	Value [n = 91]
Age [mean ± SD]	56.5 y ± 11.6 y
Gender	
Male [n, (%)]	67 (73.6%)
Female [n, (%)]	24 (26.4%)
Ancestry	
Caucasian [n, (%)]	89 (97.8%)
Other [n, (%)]	2 (2.2%)
Cirrhosis etiology	
Alcohol-related [n, (%)]	34 (37.4%)
HBV-related [n, (%)]	13 (14.3%)
HCV-related [n, (%)]	42 (46.1%)
Other causes [n, (%)]	2 (2.2%)
Child-Pugh classification	
A [n, (%)]	56 (61.5%)
B [n, (%)]	29 (31.9%)
C [n, (%)]	6 (6.6%)
Log_10_(AFP μg·L^−1^) [median (IQRs)]	0.69 (0.50–0.98)
Log_10_(SCCA-IgM AU·mL^−1^) [median (IQRs)]	1.95 (1.78–2.22)
HCC occurrences [n, (%)]	30 (33.0%)
Deaths [n, (%)]	21 (23.1%)
Liver transplantations [n, (%)]	13 (14.3%)
Dropouts [n, (%)]	22 (24.2%)

n = number, SD = standard deviation, y = years, AFP = Alpha-fetoprotein, IQR = interquartile range, μg = micrograms, L = liter, SCCA-IgM = Squamous Cell Carcinoma Antigen-Immunoglobulin M complex, AU = arbitrary units, mL = milliliter, HCC = Hepatocellular carcinoma.

### Follow-up design

Enrolled patients entered in the follow-up phase, consisting – as per typical surveillance in our center – of a clinical, biochemical and ultrasonographic re-evaluation, performed every six months for a median time of 127 months (estimated with the reverse Kaplan-Meier method). During this time, updates on clinical information, laboratory tests, imaging data and cytopathology or histopathology features (when available) were recorded. Meanwhile, all patients were treated according to the specific etiology or clinical decompensation, as recommended by the European Association for the Study of Liver guidelines. Apart from arrival to the pre-determined endpoint (November 2018), the conclusion of follow-up was also set in the event of death or liver transplantation.

### SCCA-IgM determination

Serum samples were collected in addition to routine laboratory tests, complying to a procedure which had been approved by the Ethics Committee of our Institution and was conform to the standards of the declaration of Helsinki. Levels of serum SCCA-IgM complexes were determined with a validated commercially-available ELISA kit (Hepa-IC, Xeptagen, Venice, Italy), used according to manufacturer’s instructions. The amount of SCCA-IgM was expressed in arbitrary units per milliliter (AU/mL). The intra- and inter-assay coefficients of variation resulted lower than 15%, as previously reported^23^.

### Statistical analysis

Continuous quantitative variables were expressed with means and standard deviations when normally distributed (a condition checked with Kolmogorov-Smirnov’s test), otherwise as medians and interquartile ranges. For continuous quantitative variables, comparisons among clusters were carried out either with ANOVA F test or Mann-Whitney’s test or Kruskal-Wallis’s test, depending on appropriateness. Categorical variables were expressed as frequencies and percentages: comparisons among these groups were carried out with Pearson’s Chi-square test. To analyse HCC-free survival time, we considered “HCC occurrence” as an event. To analyse overall survival time, we considered “death” and “liver transplantation” as events. To estimate the effect of considered variables on pre-defined outcomes, candidate predictors – with a limitation of the observed event-per-variable ratio ranging from five to nine – were included into a Cox proportional hazards multiple regression, operating in a stepwise backward conditional manner (entry for p < 0.05 and removal for p > 0.20). The proportional hazards assumption was checked for each predictor. For categorical prognostic variables, comparisons of survival rates were carried out with the Log-rank method. Statistical analyses were performed with the SPSS software (IBM Analytics, Armonk, NY, USA) and considered significant if p-values resulted lower than 0.05.

## Results

### Clinical and biochemical profile of the study population

Apart from age at enrollment, all the considered quantitative variables (Child-Pugh score, Alpha-fetoprotein, SCCA-IgM) displayed a non-normal distribution in the study population, hence were described through medians and inter-quartile ranges and analyzed with non-parametric tests. Missing data for serum Alpha-fetoprotein (7.7% of cases) were generated through a multiple imputation algorithm.

Because of the presence of several rightward outliers, the distribution of serum AFP and SCCA-IgM appeared highly skewed in the study population, consequently a logarithmic transformation was applied to the measured data (if unquantifiable, SCCA-IgM was approximated to 10 AU/mL, the order of magnitude of Hepa-IC’s detection limit).

Amongst genders, no differences were found in the distribution of all considered variables [results reported in Suppl. Table 1]. Similarly, no differences were found in the distribution of variables age, gender and Child-Pugh score amongst the major etiologies of cirrhosis. On the other hand, log_10_(SCCA-IgM) and log_10_(AFP) were considerably higher in hepatitis C-positive patients [results reported Suppl. Table 2].

Of the 42 HCV-infected patients enrolled in this study, 28 were only treated with an interferon-based therapy, while 14 were also treated with direct-acting antivirals.

### Analysis of the main outcomes: HCC occurrence

As synthesized in Table 2, clustering of the study population according to the development of HCC during follow-up highlighted a difference in the distribution of baseline values of log_10_(SCCA-IgM), higher in those that finally experienced HCC. These patients were also prone to suffer from a viral etiology of liver cirrhosis. Considering the outcome of HCV infected patients in relation to antiviral response, a sustained virologic response (SVR) was obtained in 5 out of 28 patients (17.9%) treated with the interferon-based regimen alone and in all the 14 patients treated with direct-acting antivirals. Among the 5 patients that achieved SVR with the “classic” interferon-based treatment, 2 patients (40.0%) developed hepatocellular carcinoma during follow-up. Among the 14 patients that achieved SVR with the more recently marketed direct-acting antivirals, one patient (7.1%) developed hepatocellular carcinoma during follow-up, while another patient (7.1%) was diagnosed with hepatocellular carcinoma during treatment itself. The limited number of patients did not allow to include antiviral treatment and/or the achievement of SVR in multivariate analysis.

**Table 2:** Comparison of baseline patient characteristics and outcomes in relation to the development of HCC during follow-up.

Variable	No HCC [n = 61]	HCC [n = 30]	p
Age [mean ± SD]	56.9 y ± 11.4 y	55.6 y ± 12.2 y	0.613^†^
Gender			0.333^‡^
Male [n, (%)]	43 (70.5%)	24 (80.0%)	
Female [n, (%)]	18 (29.5%)	6 (20.0%)	
Cirrhosis etiology			0.054^‡^
Alcohol-related [n, (%)]	28 (45.9%)	6 (20.0%)	
HBV-related [n, (%)]	8 (13.1%)	5 (16.7%)	
HCV-related [n, (%)]	23 (37.7%)	19 (63.3%)	
Other [n, (%)]	2 (3.3%)	0 (0.0%)	
Child-Pugh classification			0.588^‡^
A [n, (%)]	38 (62.3%)	18 (60.0%)	
B [n, (%)]	18 (29.5%)	11 (36.7%)	
C [n, (%)]	5 (8.2%)	1 (3.3%)	
Log_10_(AFP μg·L^−1^) [median (IQRs)]	0.67 (0.48–0.91)	0.81 (0.56–1.11)	0.080^§^
Log_10_(SCCA-IgM AU·mL^−1^) [median (IQRs)]	1.90 (1.60–2.06)	2.15 (1.91–2.70)	**0**.**004**^§^
Endpoints			**0**.**035**^‡^
Death [n, (%)]	10 (16.4%)	11 (36.7%)	
Liver transplantation [n, (%)]	7 (11.5%)	6 (20.0%)	

^†^ANOVA F test, ^‡^Pearson’s χ^2^ test, ^§^Mann-Whitney’s U test.

n = number, p = significativity, SD = standard deviation, y = years, HBV = hepatitis B virus, HCV = hepatitis C virus, AFP = Alpha-fetoprotein, μg = micrograms, L = liter, IQR = interquartile range, SCCA-IgM = Squamous Cell Carcinoma Antigen-Immunoglobulin M complex, AU = arbitrary units, mL = millililiter.

Higher levels of serum SCCA-IgM, viral etiology of liver cirrhosis and higher Child-Pugh scores emerged to be those covariates associated with a significantly greater risk of hepatocellular carcinoma development [data reported in Suppl. Table 3].

Previous reports already demonstrated that high or increasing levels of circulating SCCA-IgM were associated with increased incidence of HCC but, in order to assign a clinical meaning to the level of SCCA-IgM – which is a continuous variable – we tried to identify specific threshold values resorting to the Cutoff finder optimization bundle software^24^.

The ideal cut-off to discriminate different HCC-free survival rates in the study population was found in log_10_(SCCA-IgM) = 2.08, corresponding to SCCA-IgM = 120 AU/mL.

**Figure 1:**
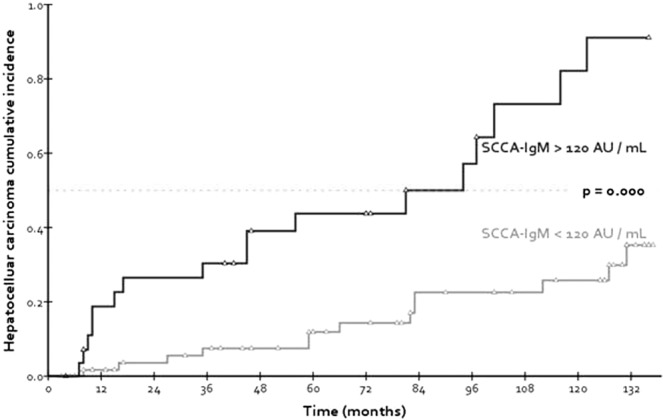
Comparison of HCC cumulative risks of the study population, here stratified by baseline levels of serum SCCA-IgM.

Applying the 120 AU/mL cut-off to baseline serum SCCA-IgM, a significant difference in HCC incidence rates emerged (Log-rank p = 0.000): this result is highlighted by the related Kaplan-Meier plot in Fig. 1. Furthermore, when baseline serum SCCA-IgM was categorized by the means of this newly identified threshold, then processed again through Cox regression, it also emerged to be an independent predictor of HCC-free survival [data reported in Suppl. Table 4]. Other “categorized” variables that appeared to contribute to the determination of a reduced HCC-free survival were: advanced liver disease (meaning Child-Pugh “B” or “C” classification) and – even if with borderline significance – viral etiology of liver cirrhosis.

### Analysis of the main outcomes: Overall survival

To estimate the effect of considered variables in the determination of survival, covariates were again processed through Cox proportional hazards regression [whose results are reported in Suppl. Table 5]. Besides Child-Pugh score, log_10_(SCCA-IgM) emerged to be a predictor of overall survival in patients with liver cirrhosis, being higher levels of serum SCCA-IgM associated with worse survival.

As previously done to distinguish HCC-free survival rates, it was determined that the best cut-off to discriminate different overall survival rates in the whole study sample was, coincidently, log_10_(SCCA-IgM) = 2.08, corresponding to SCCA-IgM = 120 AU/mL.

**Figure 2:**
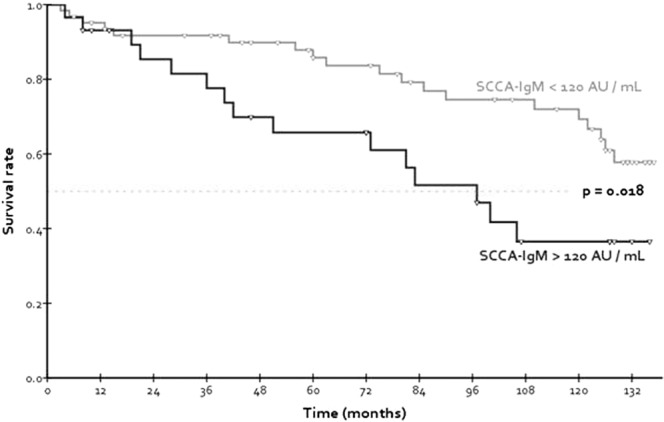
Comparison of overall survival rates of the study population, here stratified by baseline levels of serum SCCA-IgM.

Applying the 120 AU/mL cut-off to baseline serum SCCA-IgM, we confirmed the existence of a significant difference in overall survival rates (Log-rank p = 0.018), clearly appearing with the graphical representation in Fig. 2.

Baseline SCCA-IgM titer maintained the property of being a predictor of overall survival even after the categorization by the means of the newly identified cut-off. Table 3 resumes the impact on survival of the considered covariates after their transformation into categorical variables.

**Table 3:** Proportional hazards model-derived predictors of overall survival (after categorization).

Prognostic variable^†^	HR	95% CI	p
**cases = 91**, **events = 34**			
Elder age (>65 years)^‡^	1.61	0.94–2.75	0.083
High serum SCCA-IgM (>120 AU·mL^−1^)^‡^	2.14	1.29–3.57	0.003
Advanced Child-Pugh class (B–C)^‡^	2.80	1.65–4.76	0.000

^†^Omnibus test: p = 0.001 for the first step, p = 0.000 for the last step

^‡^Categorical variable.

HR = Hazard ratio, CI = Confidence interval, p = significativity, SCCA-IgM = Squamous Cell Carcinoma Antigen-Immunoglobulin M complex, AU = arbitrary units, mL = milliliter.

For those patients diagnosed with HCC, resorting to a loco-regional treatment was not associated with a significant prolongation of overall and liver transplantation-free survival [HR 0.37 (0.08–1.60), p = 0.182]. None of the considered loco-regional treatment modalities (TACE, RFA, PEI and resection) has displayed a significant overall survival benefit over the others.

Notably, the study population paralleled the expected behavior for a regular sample of patients with liver cirrhosis, being overall survival reduced in those patients with worse stages of liver disease (Log-rank p = 0.001) [representation in Suppl. Fig. 1].

### Optimization of the SCCA-IgM cut-off

The next step was to exclude the presence of biases determined by confounding variables. Through additional examination of the characteristics of the study population, it emerged that patients with higher baseline values of SCCA-IgM (>120 AU/mL) were more frequently HCV-positive and had also higher levels of serum AFP (see Table 4).

**Table 4:** Comparison of patient characteristics and outcomes in relation to the baseline titer of SCCA-IgM complex.

Variable	SCCA-IgM < 120 AU·mL^−1^ [n = 62]	SCCA-IgM > 120 AU·mL^−1^ [n = 29]	p
Age [mean ± SD]	56.5 y ± 10.3 y	56.4 y ± 14.3 y	0.984^†^
**Gender**			0.230^‡^
Male [n, (%)]	48 (77.4%)	19 (65.5%)	
Female [n, (%)]	14 (22.6%)	10 (34.5%)	
**Cirrhosis etiology**			**0**.**000**^‡^
Alcohol-related [n, (%)]	31 (50.0%)	3 (10.3%)	
HBV-related [n, (%)]	11 (17.7%)	2 (6.9%)	
HCV-related [n, (%)]	18 (29.0%)	24 (82.8%)	
Other [n, (%)]	2 (3.2%)	0 (0.0%)	
**Child-Pugh classification**			0.685^‡^
A [n, (%)]	37 (59.7%)	19 (65.5%)	
B [n, (%)]	20 (32.3%)	9 (31.0%)	
C [n, (%)]	5 (8.1%)	1 (3.5%)	
Log_10_(AFP μg·L^−1^) [median (IQRs)]	0.64 (0.45–0.87)	0.88 (0.66–1.10)	**0**.**002**^§^
HCC occurrence [n, (%)]	13 (21.0%)	17 (58.6%)	**0**.**000**^‡^
**Endpoints**			0.278^‡^
Death [n, (%)]	12 (19.4%)	9 (31.0%)	
Liver transplantation [n, (%)]	7 (11.3%)	6 (20.7%)	

^†^ANOVA F test, ^‡^Pearson’s χ^2^ test, ^§^Mann-Whitney’s U test.

pts. = patients, n = number, p = significativity, SD = standard deviation, y = years, HBV = hepatitis B virus, HCV = hepatitis C virus, AFP = Alpha-fetoprotein, μg = micrograms, L = liter, IQR = interquartile range, SCCA-IgM = Squamous Cell Carcinoma Antigen-Immunoglobulin M complex, AU = arbitrary units, mL = milliliter, HCC = hepatocellular carcinoma.

For these reasons, we decided to further process the survival rates of the study population, firstly considering HCV-positivity as stratum for the Log-rank test. The results of this process [partly represented with Suppl. Figs. 2 and 3], confirmed the existence of a significant differences in both HCC-free and overall survival rates when applying the 120 AU/mL cut-off to serum SCCA-IgM in HCV-negative patients (Log-rank p = 0.004 for HCC-free survival and Log-rank p = 0.000 for overall survival). On the other hand, this cut-off resulted unable to identify significant differences in overall survival rates in HCV-positive patients (Log-rank p = 0.033 for HCC-free survival and Log-rank p = 0.599 for overall survival). These results suggest that the cut-off for SCCA-IgM could be affected by etiology. However, because of limitations imposed by further reduction of sample numerosity, it was not possible to identify etiology-specific cut-off points in further subgroups of patients.

The performance of the same analyses with AFP as stratum was not correlated with meaningful results. Nonetheless, mortality rates tended to differ (Log-rank p = 0.050) in those patients that, besides having serum SCCA-IgM > 120 AU/mL, also displayed abnormal levels of serum AFP, suggesting an additional or complementary role for these factors.

We considered 8.8 μg/L as threshold for AFP because, according to our laboratory, it represented the upper limit of normality at the time of conduction of this study.

## Discussion

SCCA-IgM belongs to a class of molecules – the biomarkers for early HCC diagnosis – that have been evaluated in several studies^25^, but still hold an ill-defined role in clinical practice. This study was the first one to evaluate the long-term biological effects of SCCA-IgM in outpatients with liver cirrhosis.

Existing screening algorithms imprecisely assume that the risk of HCC occurrence is uniform amongst all patients with the same etiology. Instead, it appears that individual prediction of HCC risk is of paramount importance for implementing effectiveness and feasibility of screening programs^5^. For example, patients with metabolic-associated steatohepatitis will often develop HCC before the progression of liver disease to the cirrhotic stage^26^. Therefore, because of these patients with dismal progression of disease, it is advisable to re-define current recommendations for HCC screening^27^.

The results of this study offer further evidence that, irrespective of the etiology of liver cirrhosis, an important correlation amongst SCCA-IgM and the occurrence of hepatocellular carcinoma exists. For what concerns the diagnostic prediction of hepatocellular carcinoma, our data indicate the superiority of SCCA-IgM over the “classic” serological biomarker, namely Alpha-fetoprotein. Moreover, the evidences of impact on overall mortality derived from this study, suggest a potential use of serum SCCA-IgM as prognostic tool in patients diagnosed with liver cirrhosis.

During this study, liver transplantation was treated – alike death – as an “event”, because this procedure was meant to be performed in those patients with reduced life expectancy, hence censoring would have been “informative”.

Twenty two patients (24.2% of the study population) were eventually lost during follow-up. The status of drop-outs was re-checked in November 2018, resorting to our institution’s web-based application platform. It must be highlighted that these patients exclusively belonged to the CHC subgroup (where a relevant fraction decided to leave after the achievement of a sustained virologic response) and to the subgroup with alcohol-related cirrhosis (where a relevant fraction suffered from active alcohol dependency and was less motivated to comply with periodic re-evaluations).

None of the considered parameters was found to be correlated with the outcome of interest “time to liver transplantation”.

Currently, there are no studies that evaluated the behavior of SCCA-IgM in liver-transplanted subjects.

SCCA-IgM was firstly identified in HCV-positive patients. Previous reports already described an overexpression of this molecule in this condition, when compared to other causes of liver cirrhosis^18,23^. Extra-hepatic manifestations typical of chronic HCV infection – including hyper-γ-globulinemia, cryoglobulinemia, lymphoproliferative disorders and auto-antibody production – are related to B cell activation^28,29^. It is likely that easier detection of SCCA-IgM in these patients indeed relies on a deranged production of low-avidity IgM immunoglobulins.

The statistical analyses performed during this study indicate that, irrespective of the underlying etiology of liver cirrhosis, 120 AU/mL of SCCA-IgM is a threshold value above which lay those patients who are exposed to a substantially higher risk of HCC occurrence and mortality. However, when the patients were considered according to HCV-status, this cut-off point showed to maintain a fully satisfactory prognostic performance only in HCV-negative patients. Setting the cut-off at 120 AU/mL emerged to be suboptimal in HCV-positive patients (those endowed with a pronounced immune reactivity), for whom it could be suitable a higher threshold level, or normalization with other parameters. Considering that the main limitation to the search for etiology-specific cut-offs (if they exist) was determined by the limited size of the sample, further studies possibly including larger cohorts are warranted to achieve this unmet need.

Authors already described that cumulative incidence of HCC is variable with etiology, being higher in chronic viral hepatitis and lower in alcohol-induced cirrhosis^30^. Our study population was mainly constituted by patients with chronic hepatitis C, followed by patients with alcohol-related liver cirrhosis and – to a lesser extent – by HBV-infected patients (and other uncommon etiologies). Not surprisingly, viral etiology of liver cirrhosis emerged to be a predictor of reduced HCC-free survival in our sample, and most patients who developed HCC were HCV-positive.

Notably, our study population was almost exclusively composed of Caucasian people: findings may differ in other patterns of population, so an external validation is advisable.

Previous reports already identified that higher degrees of liver fibrosis and portal hypertension – conditions that are typical of an advanced stage of liver disease – are associated with increased risk of HCC development^31,32^. In this study, it emerged that advanced stage of liver disease (meaning Child-Pugh “B” or “C” class, a surrogate indicator of higher degrees of liver fibrosis and portal hypertension) was another factor found to be associated with HCC incidence rate.

At the state of the art, the behavior of SCCA-IgM and its clinical impact in HCV-infected patients that obtained a sustained virologic response with direct-acting antiviral drugs are still a matter of investigation; the same goes for the relation among long-term clinical outcomes and dynamic variations of SCCA-IgM levels during follow-up.

In conclusion, we propose the use of SCCA-IgM (with the newly identified 120 AU/mL cut-off) to help categorize patients with liver cirrhosis into different risk classes, hence guide clinical decisions like intensity of surveillance and prevention strategies.

## Supplementary information

Supplementary material.

## Data Availability

Hepa-IC is a commercially available ELISA kit, provided by Xeptagen S.p.A. Requests of provision of these patient datasets can be forwarded at any time to the corresponding Author.
