# Supplementary material for: Serum Squamous Cell Carcinoma Antigen-Immunoglobulin M complex levels predict survival in patients with cirrhosis
Source: Sci Rep. 2019 Dec 27;9:20126. doi: 10.1038/s41598-019-56633-2 (PMC6934856; doi:10.1038/s41598-019-56633-2)

SERUM SQUAMOUS CELL CARCINOMA ANTIGEN-IMMUNOGLOBULIN M COMPLEX LEVELS PREDICT SURVIVAL IN PATIENTS WITH CIRRHOSIS

Marco Cagnin[A], Alessandra Biasiolo[A], Andrea Martini[A], Mariagrazia Ruvoletto[A], Santina Quarta[A], Silvano Fasolato[A], Paolo Angeli[A], Giorgio Fassina[B] and **Patrizia Pontisso***[A]

[A] Department of Medicine, University of Padua, Italy

[B] Xeptagen S.p.A., Venice, Italy

*** Corresponding Author: Prof. Patrizia Pontisso**

E-mail: patrizia@unipd.it

Physical Address: Via Giustiniani 2, 35128 Padova (Italy)

SUPPLEMENTARY MATERIAL

Suppl. Table 1: Comparison of patient characteristics and outcomes in relation to gender

Variable	Females [n = 24]	Males [n = 67]	p
**Age [mean ± SD]**	59.25 y ± 11.87 y	55.45 y ± 11.44 y	0.17 †
**Cirrhosis aetiology**			0.22 ‡
Alcohol-related [n, (%)]	12 (50.00 %)	22 (32.84 %)	
HBV-related [n, (%)]	1 (4.17 %)	12 (17.91 %)	
HCV-related [n, (%)]	10 (41.66 %)	32 (47.76 %)	
Other [n, (%)]	1 (4.17 %)	1 (1.49 %)	
**Child-Pugh classification**			0.25 ‡
A [n, (%)]	12 (50.00 %)	44 (65.67 %)	
B [n, (%)]	9 (37.50 %)	20 (29.85 %)	
C [n, (%)]	3 (12.50 %)	3 (4.48 %)	
**Log_{10}(AFP $\mu g \cdot L^{-1}$) [median (IQRs)]**	0.72 (0.38 – 0.99)	0.68 (0.52 – 0.96)	0.80 §
**Log_{10}(SCCA-IgM $AU \cdot mL^{-1}$) [median (IQRs)]**	1.93 (1.77 – 2.38)	1.96 (1.79 – 2.21)	0.95 §
**HCC occurrence [n, (%)]**	6 (25.00 %)	24 (35.82 %)	0.45 ‡
**Endpoints**			0.84 ‡
Death [n, (%)]	7 (29.17 %)	14 (20.90 %)	
Liver transplantation [n, (%)]	3 (12.50 %)	10 (14.93 %)	
**Dropouts [n, (%)]**	6 (25.00 %)	16 (23.88 %)	0.91 ‡

† ANOVA F test, ‡ Pearson's χ^2 test, § Mann-Whitney's U test

pts. = patients, n = number, p = significativity, SD = standard deviation, y = years, HBV = hepatitis B virus, HCV = hepatitis C virus, AFP = Alpha-fetoprotein, µg = micrograms, L = liter, IQR = interquartile range, SCCA-IgM = Squamous Cell Carcinoma Antigen-Immunoglobulin M complex, AU = arbitrary units, mL = milliliter, HCC = hepatocellular carcinoma

Suppl. Table 2: Comparison of patient characteristics and outcomes in relation to the main aetiologies of liver cirrhosis

Variable	Alcohol-related (n = 34)	HBV-related (n = 13)	HCV-related (n = 42)	p
**Age [mean ± SD]**	57.68 y ± 9.42 y	54.46 y ± 12.38 y	55.81 y ± 13.20 y	0.65 †
**Gender [n, (%)]**				0.14 ‡
Male [n, (%)]	22 (64.71 %)	12 (92.31 %)	32 (76.19 %)	
Female [n, (%)]	12 (35.29 %)	1 (7.69 %)	10 (23.81 %)	
**Child-Pugh classification**				0.08 ‡
A [n, (%)]	16 (47.06 %)	8 (61.54 %)	30 (71.43 %)	
B [n, (%)]	13 (38.23 %)	4 (30.77 %)	12 (28.57 %)	
C [n, (%)]	5 (14.71 %)	1 (7.69 %)	0 (0.00 %)	
**Log_{10}(AFP $\mu g \cdot L^{-1}$) [median (IQRs)]**	0.68 (0.47 – 0.77)	0.60 (0.41 – 0.75)	0.90 (0.61 – 1.19)	**< 0.01** §
**Log_{10}(SCCA-IgM $AU \cdot mL^{-1}$) [median (IQRs)]**	1.80 (1.58 – 1.94)	1.85 (1.53 – 2.06)	2.19 (1.96 – 2.71)	**< 0.01** §
**Undosable serum SCCA-IgM [n, (%)]**	4 (11.76 %)	2 (15.39 %)	1 (2.38 %)	0.18 ‡
**HCC occurrence [n, (%)]**	6 (17.65 %)	5 (38.46 %)	19 (45.24 %)	**0.04** ‡
**Endpoints**				0.64 ‡
Death [n, (%)]	8 (23.53 %)	2 (15.39 %)	11 (26.19 %)	
Liver transplantation [n, (%)]	4 (11.76 %)	4 (30.77 %)	5 (11.91 %)	
**Dropouts [n, (%)]**	10 (29.41 %)	0 (0.00 %)	12 (28.57 %)	0.08 ‡

† ANOVA F test, ‡ Pearson's χ^2 test, § Kruskal-Wallis's test

HBV = hepatitis B virus, HCV = hepatitis C virus, n = number, p = significativity, SD = standard deviation, y = years, AFP = Alpha-fetoprotein, µg = micrograms, L = liter, IQR = interquartile range, SCCA-IgM = Squamous Cell Carcinoma Antigen-Immunoglobulin M complex, AU = arbitrary units, mL = milliliter, HCC = hepatocellular carcinoma

Suppl. Table 3: Proportional hazards model-derived predictors of HCC-free survival (before categorization)

cases = 87, events = 30			
**Prognostic variable** †	**HR**	**95 % CI**	**p**
**Log_{10}(serum SCCA-IgM)**	2.39	1.09 – 5.13	**0.03**
**Viral aetiology** ‡	2.55	1.12 – 5.80	**0.03**
**Child-Pugh score**	1.34	1.06 – 1.70	**0.01**

† *Omnibus test: p = 0.005 for the first step, p = 0.000 for the last step*
‡ *Categorical variable*

HCC= hepatocellular carcinoma, HR = hazard ratio, CI = confidence interval, p = significativity, SCCA-IgM = Squamous Cell Carcinoma Antigen-Immunoglobulin M complex

Suppl. Table 4: Proportional hazards model-derived predictors of HCC-free survival (after categorization)

cases = 87, events = 30			
**Prognostic variable** †	**HR**	**95 % CI**	**p**
**Viral aetiology** ‡	2.00	0.95 – 4.19	0.07
**Advanced Child-Pugh class (B – C)** ‡	2.15	1.24 – 3.73	**≤ 0.01**
**High serum SCCA-IgM (> 120 AU·mL^{-1})** ‡	2.62	1.45 – 4.73	**≤ 0.01**

† *Omnibus test: p = 0.000 for the first step, p = 0.000 for the last step*
‡ *Categorical variable*

HCC= hepatocellular carcinoma, HR = hazard ratio, CI = confidence interval, p = significativity, SCCA-IgM = Squamous Cell Carcinoma Antigen-Immunoglobulin M complex, AU = arbitrary units, mL = milliliter

Suppl. Table 5: Proportional hazards model-derived predictors of overall survival (before categorization)

cases = 91, events = 34			
**Prognostic variable** †	**HR**	**95 % CI**	**p**
**Age**	1.02	0.99 – 1.05	0.17
**Log_{10}(serum SCCA-IgM)**	2.78	1.37 – 5.62	**< 0.01**
**Child-Pugh score**	1.28	1.10 – 1.50	**< 0.01**

† *Omnibus test: p = 0.001 for the first step, p = 0.000 for the last step*

HR = hazard ratio, CI = confidence interval, p = significativity, SCCA-IgM = Squamous Cell Carcinoma Antigen-Immunoglobulin M complex

Suppl. Figure 1: Comparison of survival rates of the study sample, here stratified by a renowned prognostic score (Child-Pugh classification "A" versus "B" combined with "C")

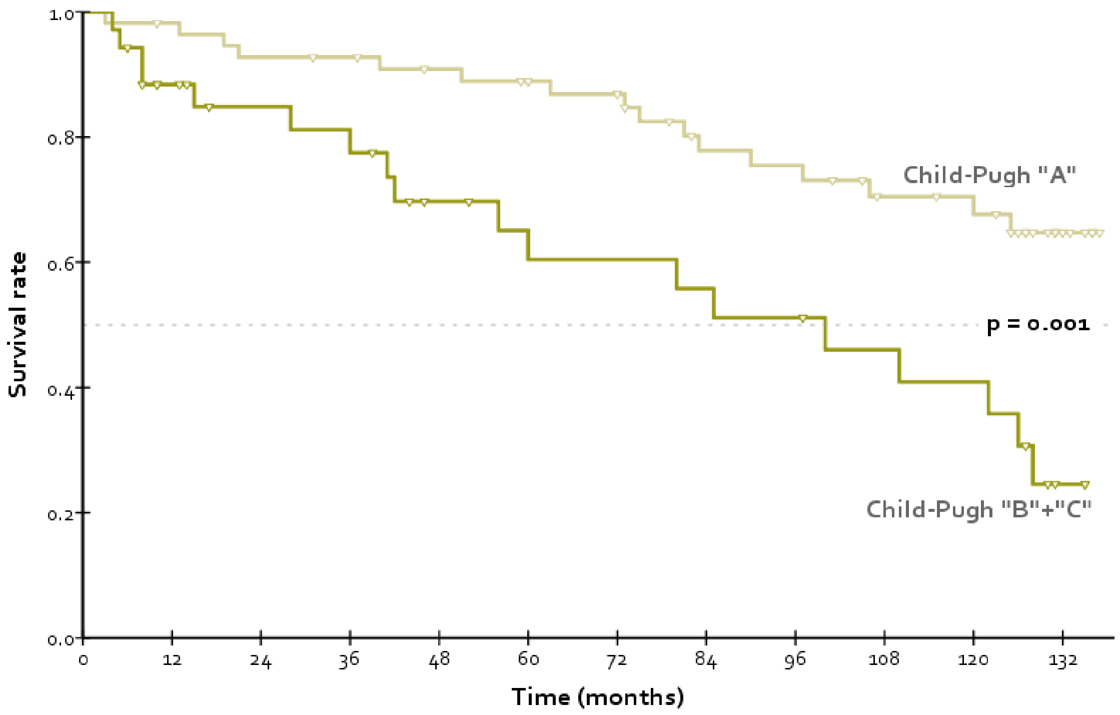

Suppl. Figure 2: Comparison of HCC-free survival rates of the study sample, here stratified by baseline levels of serum SCCA-IgM in HCV-negative (up) and HCV-positive (down) patients.

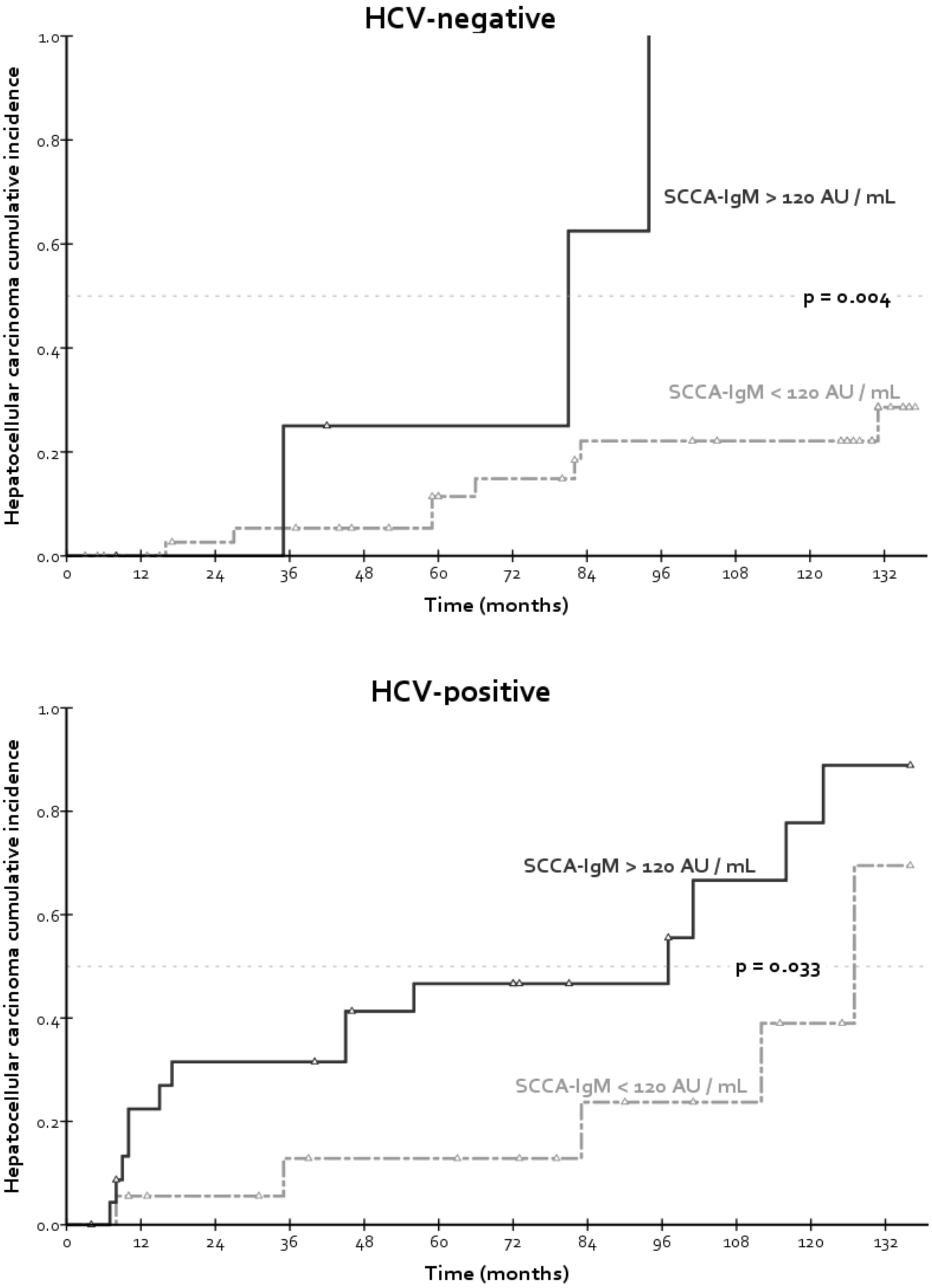

Suppl. Figure 3: Comparison of overall survival rates of the study sample, here stratified by baseline levels of serum SCCA-IgM in HCV-negative (up) and HCV-positive (down) patients.

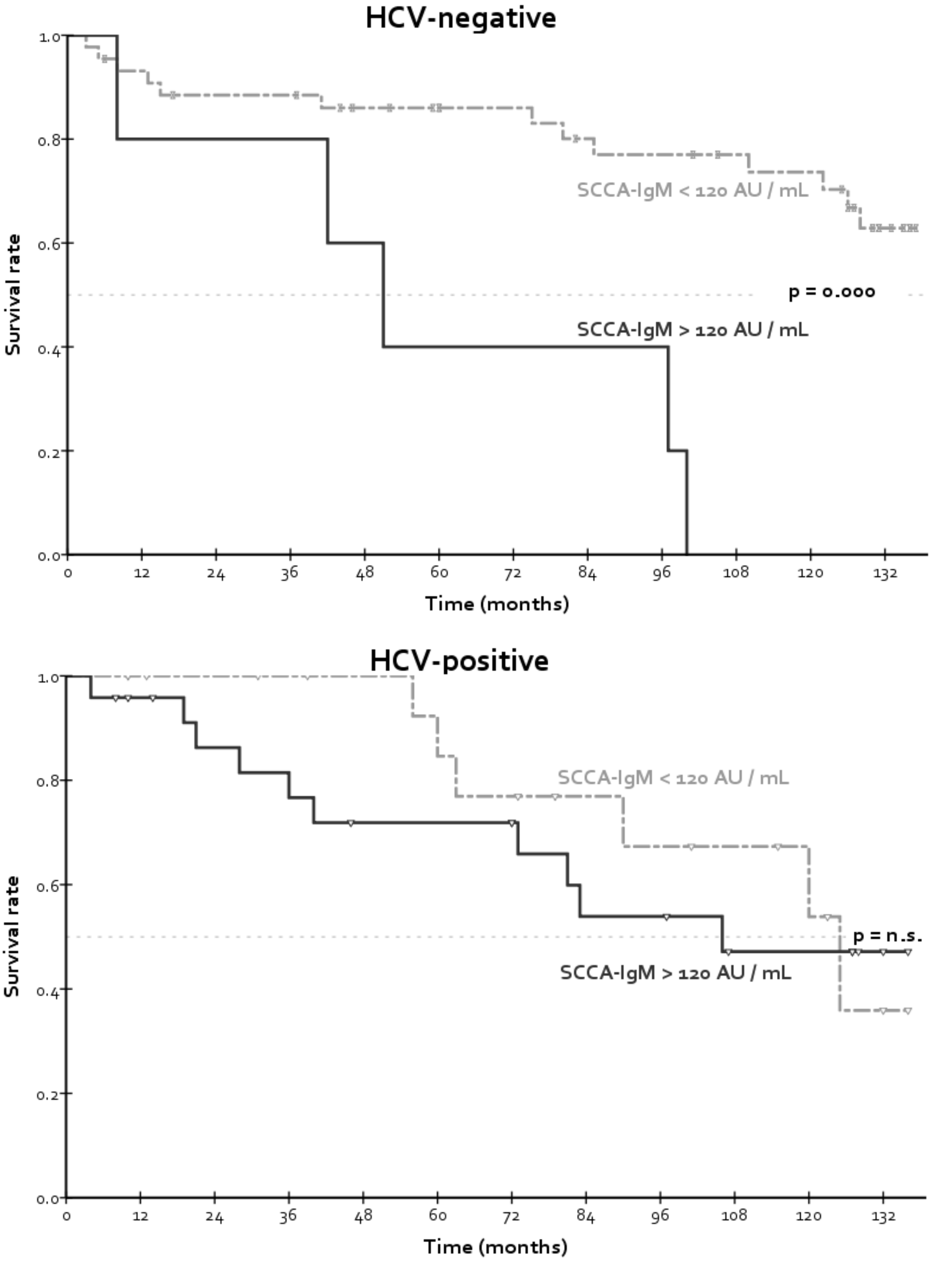

Supplement: Supplementary file 1 — Supplementary material. [file 41598_2019_56633_MOESM1_ESM.pdf]
